# Single-Crystal X-ray Structures of conductive π-Stacking Dimers of Tetrakis(alkylthio)benzene Radical Cations

**DOI:** 10.1038/srep29314

**Published:** 2016-07-11

**Authors:** Xiaoyu Chen, Feng Gao, Wuqin Yang

**Affiliations:** 1 School of Safety, China University of Mining and Technology, Xuzhou 221008, P. R. China; 2 State Key Laboratory of Coordination Chemistry, Nanjing National Laboratory of Microstructures, Nanjing University, Nanjing 210093, P. R. China; 3 School of Chemistry and Chemical Engineering, Jiangsu Key Laboratory of Green Synthetic Chemistry for Functional Materials, Jiangsu Normal University, Xuzhou 221116, P. R. China

## Abstract

Salts containing radical cations of 1,2,4,5-tetrakis(isopropylthio)benzene (TPB) and 1,2,4,5-tetrakis(ethylthio) benzene (TEB) have been successfully synthesized with 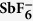. These newly synthesized salts have been characterized by UV-Vis absorption, EPR spectroscopy, conductivity measurement, single crystal X-ray diffraction analysis as well as DFT calculation. This study raises the first crystal structure of conductive π-stacking radical cation with single phenyl ring and reveals their conductivity has relationship with the stack structure which affected by the substituent.

Infinite face-to-face π-stacking formations are known to be important for electrical and magnetic conduction in charge-transfer (CT) complex^1^,^2^,^3^,^4^,^5^,^6^, since research reveals electron can migrate across the chains in form of unpaired electron interchain transport^7^,^8^. Organic conjugated oligomers assembled into π-stacks are assumed to be conductive if they could relay reaction via the stacks^9^. The crystallization of π-conjugated co-oligomer radical cation 3′,4′-dibutyl-5,5′′-diphenyl-2,2′:5′,2′′-terthiophene in 1996 gave the first example of these π-stacked conductive materials (Fig. 1)^10^. Owing to its intrinsic instability and difficulty of crystallization, no second example of π-stacked oxidized conjugated oligomers has been structurally characterized until we stabilized the conductive crystals of 4,4′-terphenyldiamine radical cations in 2012 by introducing the large π-conjugate system and weak coordinating anion (WCA) (Fig. 1)^11^. It is known that π-radical cations can be made persistently stable and isolated by appropriate structural modification with π-conjugated systems or alkyl substituents^12^. Steric protection with alkyl or alkoxy groups could lead to stable and soluble (co-)oligomer radical cations, but at the same time is undesirable for formation of infinite π-stacks, and hence undesirable for electrical conduction^11^. Therefore, the conductive π-stacked radical cation without large π-conjugated system has not been stabilized yet.

On the other hand, radical cations contained S atoms that play vital roles in chemical mechanism, biochemistry and material science have attracted considerable attention^13^,^14^,^15^,^16^. π-Stacks of π-conjugated radical cations with thio groups can be potential applications to molecular electronics^17^. CT complex consisted of electron donor tetrathiafulvalene (TTF) radical cation and electron acceptor 7,7,8,8-tetracyanoquino-dimethane (TCNQ) radical anion is a most notable example^18^,^19^,^20^. Conductive thiophene/phenylene co-oligomer and TTF radical cations have also been stabilized by WCA recently (Fig. 1)^21^,^22^,^23^,^24^. These dramatic results led by S atoms enlighten us to study on the single phenyl substituted with alkylthio groups.

We now report syntheses, crystal structures, spectroscopic characterizations, and physical properties of 1,2,4,5-Tetrakis-(isopropylthio)benzene (TPB) and 1,2,4,5-Tetrakis(ethylthio)-benzene (TEB) radical cations with weakly coordinating anion 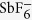. Our results give first example of π-stacking conductive radical cation with single phenyl ring. The study of CT complex can be less dependent on the hard-soluble oligomers.

## Results

### Synthesis

**Figure 2:**
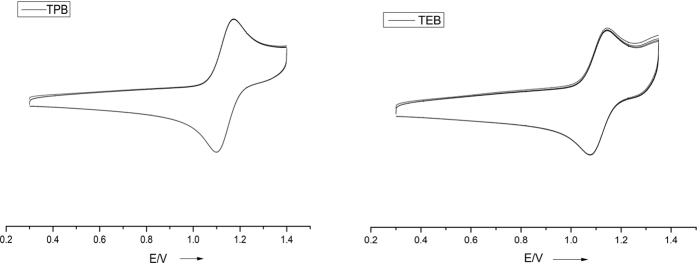
Cyclic voltammograms of TPB and TEB in CH_2_Cl_2_ (2 × 10^−4^ M, 0.1 M n-NBu_4_PF_6_) were measured at 50 mV/s at 288 K. (E^TEB^_ox_ = 1.17 V, E^TPB^_ox_ = 1.18 V, vs Ag/Ag^+^; working electrode: platinum, reference electrode: Ag/AgCl, and counter electrode: platinum wire; Scanning direction is from negative to positive).

TPB was commercially available while TEB was prepared according to the literature^25^,^26^. Cyclic voltammetry of them in CH_2_Cl_2_ at room temperature with 0.1 M NBu_4_PF_6_ as the supporting electrolyte showed well-defined curves with oxidation peaks at 1.17 (TEB) and 1.18 V (TPB, vs Ag/Ag^+^), respectively (Fig. 2), indicating that the radical cations are stable under these conditions. Upon oxidation with NOSbF_6_, TPB and TEB successfully converted to soluble radical cation salts (Eq. 1). 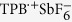 and 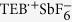 were formed by oxidized TPB and TEB from equivalent NOSbF_6_, while 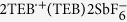 was obtained from excess amount of TEB.


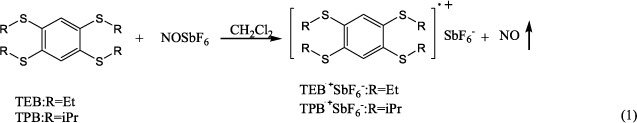


### Crystal structures

**Figure 3:**
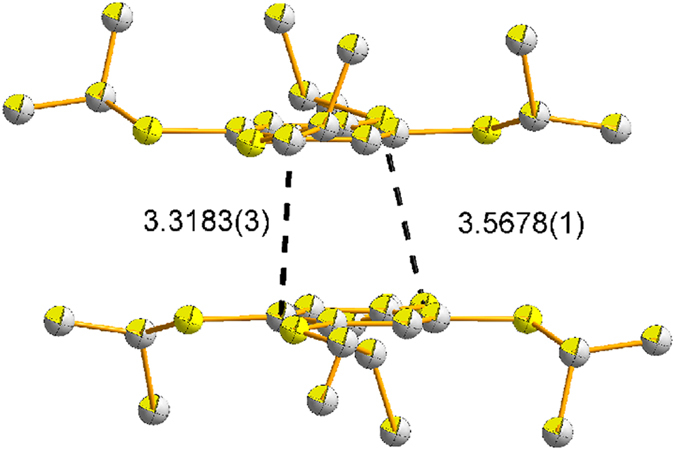
Structure of the π-dimer moity in TPB^·+^ showing intermolecular interactions within dimers (distance [Å], hydrogen atoms are not shown).

**Figure 4:**
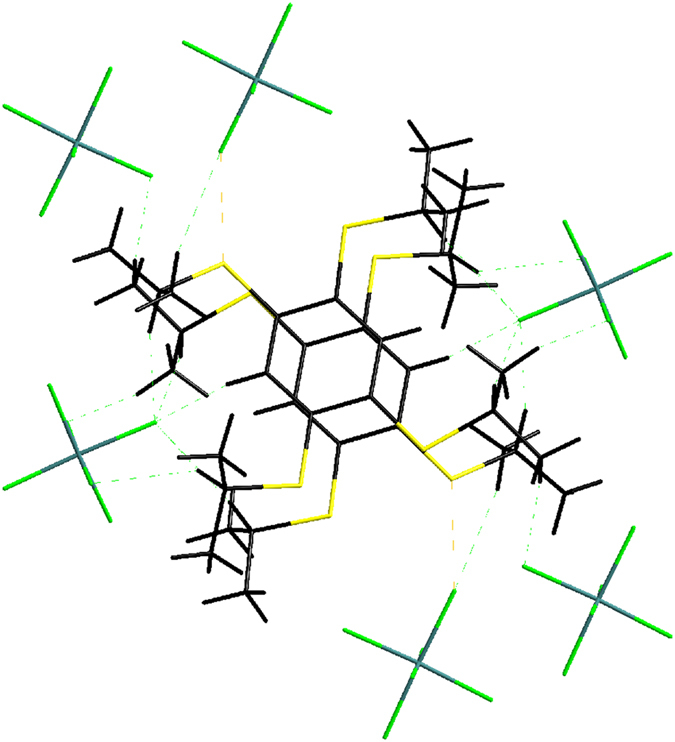
Crystal packing of 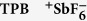.

**Figure 5:**
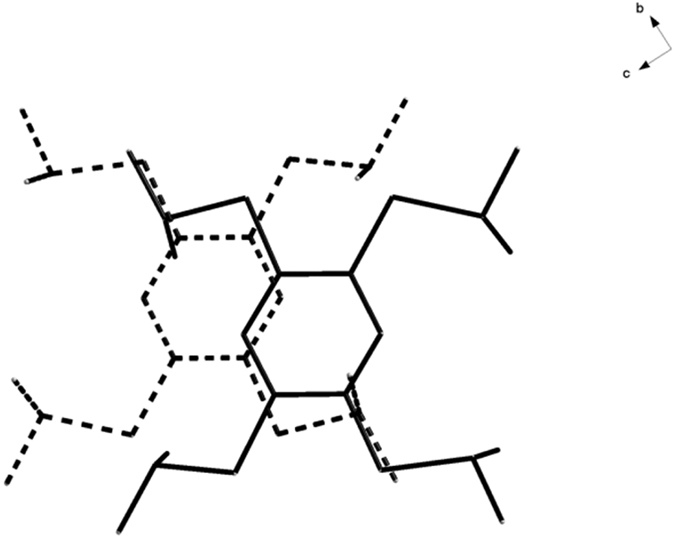
A stereoview of the crystal structure of TPB^·+^ from *a* axis.

**Figure 6:**
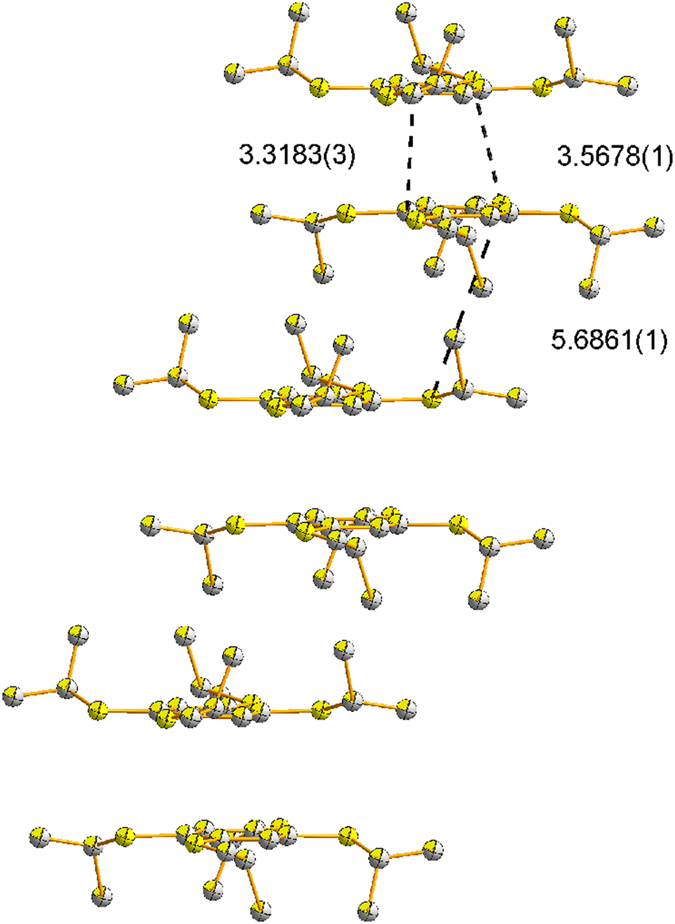
Stereoscopic view of the stack of radical cation TPB^·+^(distance [Å], hydrogen atoms are not shown).

Blue block crystals suitable for X-ray crystallographic studies were obtained from the dichloromethane solution of 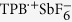 at −20 °C, and the details of the crystallographic data are shown in support information (Tables S1, S2, SI). The single crystal X-ray diffraction reveals that TPB^**·**+^ has π-dimer structure (Fig. 3). Dimeric pairs are separated by counter anions. Cations and anions are linked by considerable H…F and S…F contacts (Fig. 4). One stack is neighboring to six others by aromatic-aromatic interactions (3.6898(7) Å; Fig. 4). Close packings of the TPB^**·**+^ in dimeric pairs lead to staggered arrangements of the iPr groups to avoid steric crowding (Fig. 5). In a π-dimer, two independent radical cations have essentially the same structures (Fig. 3). The closest interplanar C-C distance (3.3183(3) Å) and S-S (3.5678(1) Å) between the two radical cations in the dimeric pairs are less than the sum of the van der Waals radii of these atoms (C-C 3.40 Å; S-S 3.70 Å) respectively, indicating electronic couplings between them. However, the closest interdimeric S-S distance (5.6861(1) Å) is too long, thus no interaction is observed between the pairs (Fig. 6).

**Figure 7:**
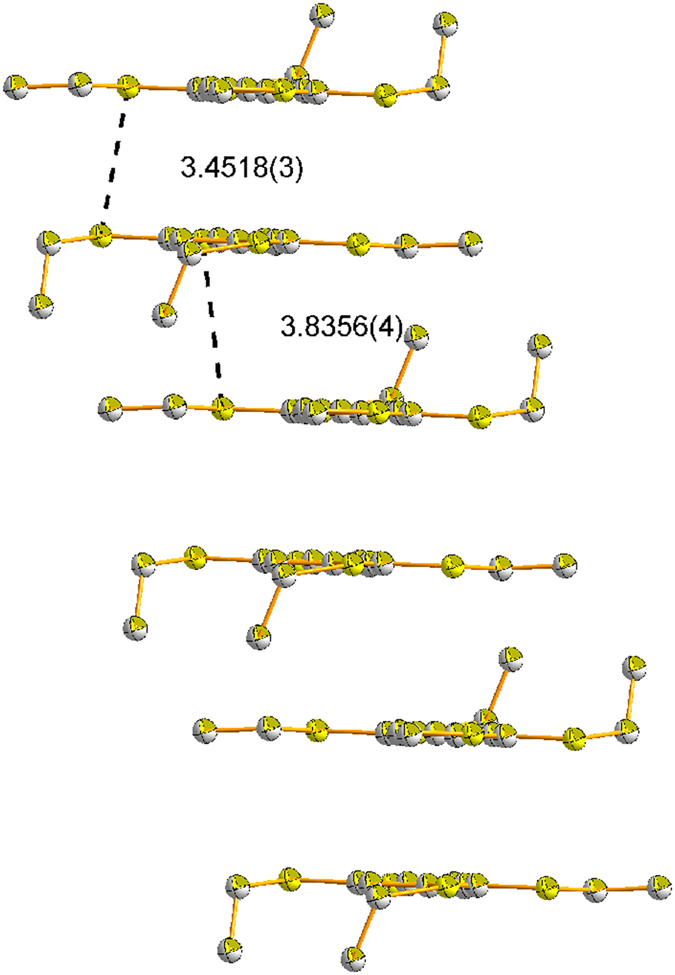
Stereoscopic view of the stack of radical cation TEB^·+^(distance [Å], hydrogen atoms are not shown).

The blue needle-like crystals cooling from the solution obtained from equivalent TEB and NOSbF_6_ have totally different structure compared with 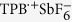 (Fig. 7). 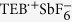 stacks along *a* axis as dimers and crystallizes in the space group *P-1* (Tables S1, S2; Figures S1, S2, SI). In a stack, the closest interplanar S-S distance between the two radical cations is 3.4518(3), indicating the electron can migrate well between them, while the closest interdimeric S-S distance (3.8356(4) Å) of two neighboring dimers is a little longer than 3.70 Å, implying a weak electronic couplings.

**Figure 8:**
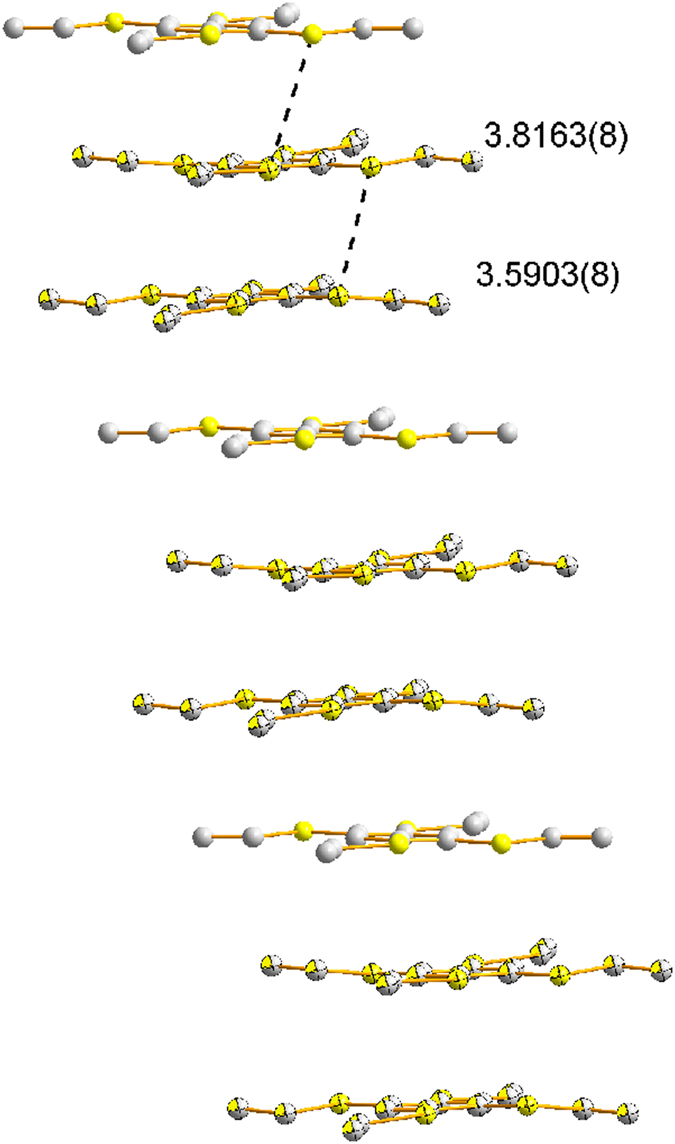
Stereoscopic view of the stack of radical cation 2TEB^·+^(TEB) (distance [Å], hydrogen atoms are not shown).

Crystals of 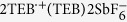 were obtained by using excess amount of TEB. The single crystal X-ray diffraction demonstrated neutral TEBs inserted in the stacks (Fig. 8, Tables S1, S2; Figures S3, S4, SI). As shown, TEB^**·**+^ stacks as dimers and between two dimers there is a neutral TEB (Table S2, SI)^14^,^26^, the dimers and the neutral molecules are arranged at regular intervals. The closest interplanar S-S distance (3.5903(8) Å) between the two radical cations in the dimeric pairs is shorter than the sum of the van der Waals radii of a sp^2^ S-S interaction (3.70 Å), indicating strong electronic couplings between them. However, the closest S-S distance (3.8163(3) Å) between one radical cation and one neutral molecule is longer than 3.70 Å. The insulation of the neutral TEB make the electron could not migrate well across the neutral molecule. Therefore, the π-reaction could not convey thoroughly via the stacks.

### UV-Vis and EPR spectroscopic analyses

**Figure 9:**
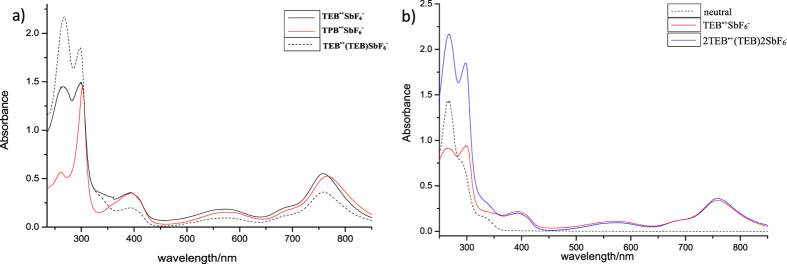
(**a**) Absorption spectra of 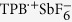, 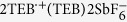 and 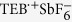; (**b**) Absorption spectra of neutral TEB, 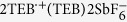 and 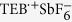 (10^−5^ M in CH_2_Cl_2_ at 25 °C).

**Figure 10:**
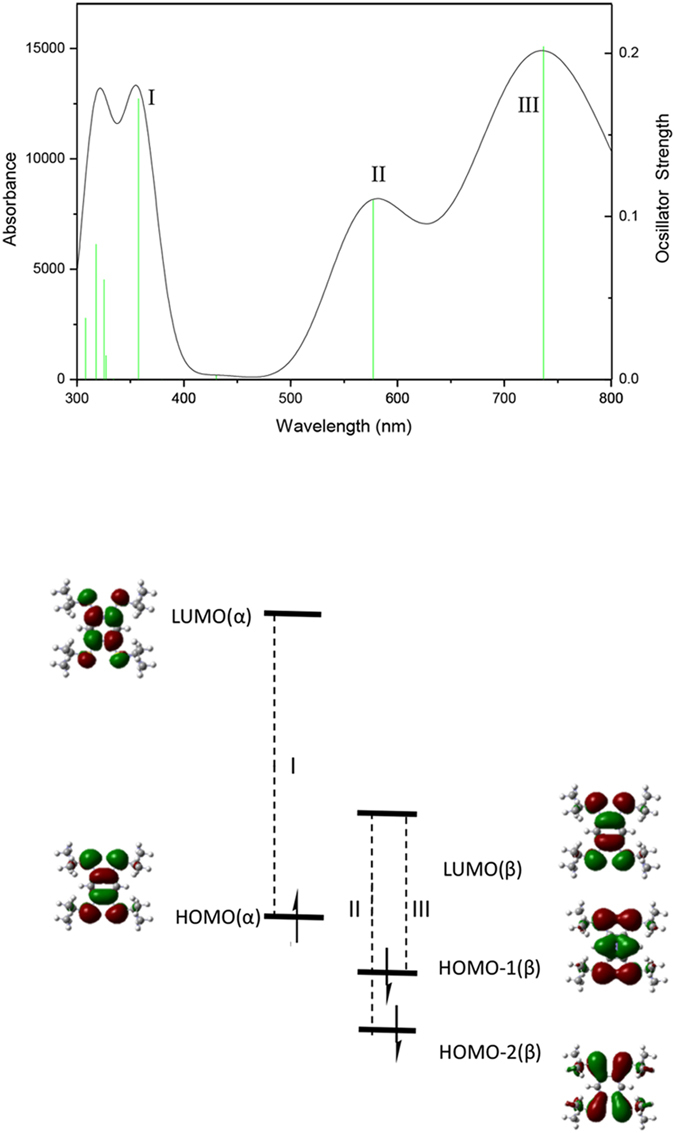
(Top) UV absorption spectrum of TPB^·+^ calculated at the (U)B3LYP/6-31+G(d) level. The spectra were simulated by using a Lorentzian convolution with 500 cm^−1^ half-widths; (Bottom) Frontier molecular orbitals and electronic transitions of TPB^**·**+^.

UV-Vis absorption spectra of 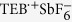, 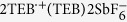 and 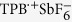 are presented in Fig. 9a 27, while the spectra of 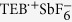, 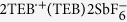 and neutral TEB are in Fig. 9b. As seen in Fig. 9a, spectra of 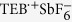 and 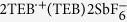 have the same peak position, while the spectrum of 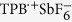 shows a slight red shift. However, the absorption intensity of 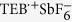 and 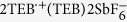 below 350 nm is different, the absorption intensity of 2TEB^**·**+^(TEB)2SbF_6_ is supposed to be sum of 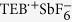 and TEB in Fig. 9b. To qualitatively assign electronic transitions in the UV-Vis spectra of these radical cations, we carried out time-dependent (TD)DFT computations at the (U)B3LYP/6-31+G(d,p) levels using Gaussian [Frisch, M. J. *et al*. Gaussian 09, revision B.02 (Gaussian, Inc., Wallingford CT, 2010)]. The calculated transition energies of monomeric radical cations are in qualitative agreement with the experimental UV-Vis spectra, and here, only the results for TPB^**·**+^ are illustrated in Fig. 10. The peak sat around 760 nm, 580 nm and 390 nm are assigned to the transitions of HOMO(α)→LUMO(α) (**I**), HOMO(β)-2→LUMO(β) (**II**), and HOMO(β)-1→LUMO(β) (**III**), respectively (The calculated transitions from 357 nm to 308 nm are not shown). Transitions in the UV spectrum of TEB^**·**+^ (Figures S5, SI) are similar to those of TPB^**·**+^.

**Figure 11:**
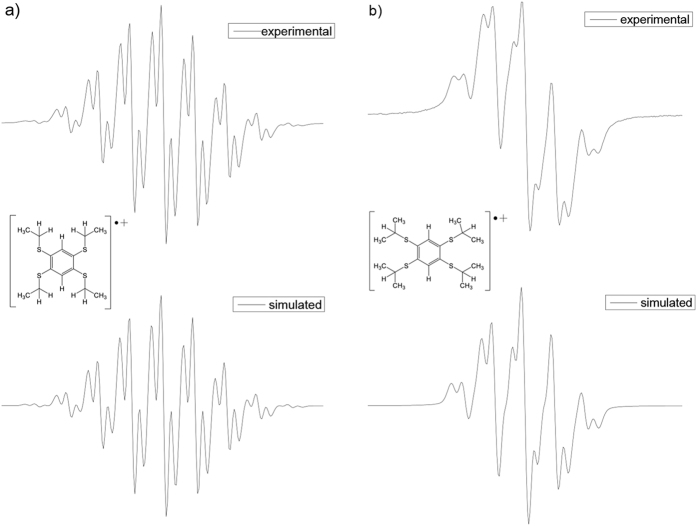
(**a**) Experimental (CH_2_Cl_2_, 1 × 10^−4^ M, 233 K) and simulated EPR spectra of TEB^**·**+^ with 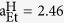 G (8H), 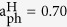 G (2H), g = 2.0076; (**b**) Experimental (CH_2_Cl_2_, 1 × 10^−4 ^M, 233 K) and simulated EPR spectra of TPB^**·**+^ with 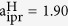 G (8H), 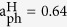 G (2H), g = 2.0072.

**Figure 12:**
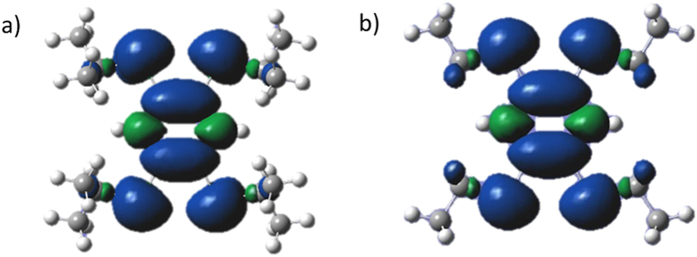
Spin-density maps for TPB^**·**+^ (**a**) and TEB^**·**+^ (**b**) calculated with the (U)B3LYP/6-31+G(d, p) method. The spin density was drawn at the isovalue of 4 × 10^−4^ e/bohr^3^.

The EPR spectrum of solution samples (Fig. 11) gave a powerful support of the formation of the tetrakis(akylthio)benzene radical cations. The hyperfine coupling of hydrogen atoms in the solution EPR spectra are in good agreement with the patterns resulting from interactions with H_ring_ (2H) and H_akyl_ (SEt or SiPr) atoms^1^. This can be rationalized as the single charge is stabilized by the whole molecule seen from the experimental and simulated results as well as the calculated spin density maps (Fig. 12)^28^. 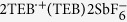 behaves the same as 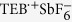 in EPR spectrum because neutral TEB has no signal.

### Conductivity measurements

**Figure 13:**
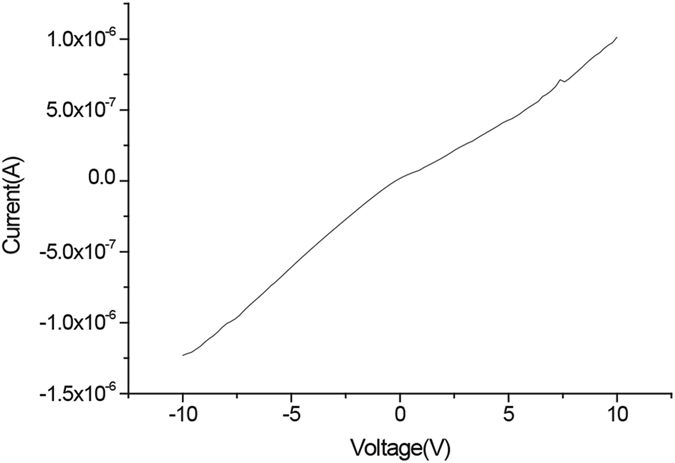
I-V curve for 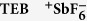 at room temperature.

Two-probe single-crystal conductivity measurements on 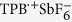, 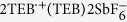 and 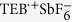 at room temperature gave σ = 3.9 × 10^−7^ S/cm, 2.04 × 10^−6^ S/cm, and 1.09 × 10^−5^ S/cm along the axises on which stacks lie, respectively (Fig. 13, Table 1). Spin density maps showed the electron cloud on S atoms contribute a lot to the delocalization of charge. It is known that the conductivity of π-stacked radical cations originates from the migration of π-interactions between chains while anions have no contribution to conductivity^11^. It can be expected that conductivity would be higher along the stacking direction (i.e., through π-stacks) because the axis of the stacking directions are not overlap with the axises of the unit cells (This can be seen from the stereoviews of the crystal structures in Fig. 4, Figures S2 and S4, SI)^10^,^27^. Comparing 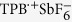 and 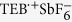, we noticed the conductivity was improved by reducing steric hindrance of the substituents. 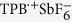 gave the lowest conductivity because of its long interdimeric distance, from which the π-dimers were entirely separated. Comparing 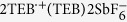 and 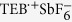, we could see the neutral molecule is an obstacle for electron transfer in a stack even though electron could go through its π-electron cloud^29^,^30^.

## Conclusions

In conclusion, radical cations of 1,2,4,5-tetrakis(isopropylthio)benzene (TPB) and 1,2,4,5-tetrakis(ethylthio)benzene (TEB) have been successfully isolated as stable crystals, and their structures were determined by X-ray crystallography. Upon oxidation with NOSbF_6_, TPB and TEB form stacks of radical cation π-dimers. Conductivity measurement reveals the interdimeric distance between two dimer pairs determines the ability of charge transformation. Thus our work not only raise the first example of π-stacking radical cation with single phenyl ring of weak conductivity, but also lead to a steric control synthesis and provide a systematic study of conductive π-stacking radical cations. Isolation of such radical species together with their structural determination will open up a new avenue for electrical conductors^21^.

## Methods

### General Procedures

All experiments were carried out under a nitrogen atmosphere using standard Schlenk techniques and a glove box. 1,2,4,5-tetrakis(isopropyl-thio)benzene (TPB) and NOSbF_6_ (Alfa Aesar) were purchased and used upon arrival. 1,2,4,5-tetrakis(ethylthio)benzene (TEB) were prepared according to literatures^25^. Solvents were dried prior to use. EPR spectra were obtained using Bruker EMX-10/12 at room temperature. UV-Vis spectra were recorded on Lambda 35 spectrometers. Element analyses were performed on Elementar Vario EL III at Shanghai Institute of Organic Chemistry, the Chinese Academy of Sciences. X-ray crystal structures were obtained by Bruker APEX-II CCD and PHOTON100 CMOS detectors. Single crystals were coated with Paratone-N oil and mounted using a glass fiber. Crystal data and structure refinement details are listed in the supporting information (Table 1, SI). For conductivity measurements, single-crystal samples were affixed on glass carriers and silver paste was used to connect samples and electrodes along the crystallogarphic axises. I-V curves were measured by using a computer-controlled Keithley 2400 source meter.

### Synthesis of 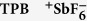, 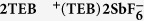 and 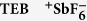


#### 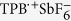


Under anaerobic and anhydrous conditions, a mixture of TPB (0.100 g, 0.27 mmol), and NOSbF_6_ (0.071 g, 0.27 mmol) in CH_2_Cl_2_ (~50 ml) were stirred at room temperature for 1d. The resultant blue solution was filtered. The filtrate was then concentrated and stored at ca. −20 °C for 1d to afford X-ray-quality crystals of radical salt 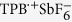. Yield: 0.12 g, 73%; Elemental analysis (%): calcd C 35.42, H 4.95; found C 35.71, H 5.10.

#### 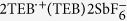 

TEB (0.127 g, 0.40 mmol), and NOSbF_6_ (0.071 g, 0.27 mmol). Yield: 0.13 g, 68%; Elemental analysis (%): calcd C 35.31, H 4.62; found C 35.24, H 4.60.

#### 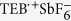 

TEB (0.086 g, 0.27 mmol), and NOSbF_6_ (0.071 g, 0.27 mmol). Yield: 0.11 g, 74%; Elemental analysis (%): calcd C 30.33, H 4.00; found C 29.78, H 4.36.

## Additional Information

**How to cite this article**: Chen, X. *et al*. Single-Crystal X-ray Structures of conductive π-Stacking Dimers of Tetrakis(alkylthio)benzene Radical Cations. *Sci. Rep.* **6**, 29314; doi: 10.1038/srep29314 (2016).

## Supplementary Material

Supplementary Information

## Figures and Tables

**Figure 1:**
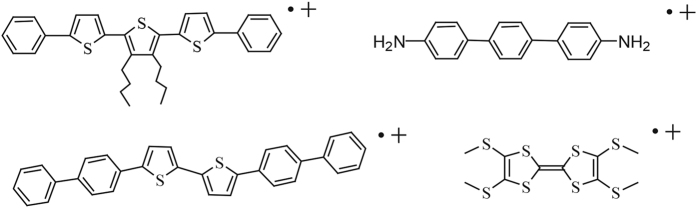
Conductive *π*-conjugated (co-)oligomer radical cations.

**Table 1:** Conductivity of crystals of radical cations.

Name	Conductivity, σ (S/cm, ×10^−6^)	Axis
TPB^**·**+^	0.39	*a* axis(100)
2TEB^**·**+^(TEB)2Sb 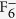	2.04	*b* axis(010)
TEB^**·**+^	10.9	*a* axis(100)
