# Supplementary material for: Single-Crystal X-ray Structures of conductive π-Stacking Dimers of Tetrakis(alkylthio)benzene Radical Cations
Source: Sci Rep. 2016 Jul 11;6:29314. doi: 10.1038/srep29314 (PMC4941407; doi:10.1038/srep29314)
Supplement: Supplementary Information [file srep29314-s1.pdf]

Supporting Information for

Single-Crystal X-ray Structures of conductive π-Stacking Dimers of Tetrakis(alkylthio)benzene Radical Cations

Xiaoyu Chen[1,3], Feng Gao[2], Wuqin Yang[1]

[1] School of Safety, China University of Mining and Technology, Xuzhou 221008, P.R. China
[2] School of Chemistry and Chemical Engineering, Jiangsu Key Laboratory of Green Synthetic Chemistry for Functional Materials, Jiangsu Normal University, Xuzhou 221116, P. R. China
[3] State Key Laboratory of Coordination Chemistry, Nanjing National Laboratory of Microstructures, Nanjing University, Nanjing 210093, P.R. China

Contents

Table S1. Crystallographic Data for All of the Compounds………………………………………… S2

Table S2. Selected Bond Lengths (Å) for All of the Compounds…………………………….….... S2

Figure S1. Structure of the stack of $TEB^{•+}$…………………………………………….………….……S3

Figure S2. A stereoview of the crystal structure of $TEB^{•+}$ from *a* axis………….......................... S3

Figure S3. Structure of the stack of $2TEB^{•+}$(TEB) …………………………………………………S4

Figure S4. A stereoview of the crystal structure of $2TEB^{•+}$(TEB) from *b* axis............................ S4

Figure S5. Calculation of the UV absorption spectra of $TEB^{•+}$…………………………….……S5

Table 1. Crystallographic Data for All of the Compounds

	**$TPB^{\bullet+}SbF_6^-$,**	**$TEB^{\bullet+}SbF_6^-$**	**$2TEB^{\bullet+}(TEB)2SbF_6^-$**
**formula**	$C_{18}H_{30}S_4SbF_6$	$C_{14}H_{22}S_4SbF_6$	$C_{42}H_{66}S_{12}Sb_2F_{12}$
**fw**	610.41	554.31	1427.17
**cryst syst**	Monoclinic	Triclinic	Monoclinic
**space group**	P2(1)/n	P-1	P2(1)/n
**a, Å**	9.2302(8)	7.6683(8)	10.5048(13)
**b, Å**	19.2894(16)	13.2164(14)	11.1119(14)
**c, Å**	14.3108(12)	20.0113(17)	24.429(3)
**α, deg**	90.00	90.113(3)	90.00
**β, deg**	19.2894(16)	90.349(3)	96.3930(10)
**γ, deg**	90.00	91.773(4)	90.00
**V, Å3**	2486.7(4)	2027.1(3)	2833.8(6)
**Z**	4	4	2
**ρcalcd, g cm−3**	1.630	1.816	1.673
**T/K**	123(2)	123(2)	123(2)
**μ, mm−1**	1.493	1.822	1.466
**θ, deg**	1.8-27.58	3.03-25.16	2.21-27.35
**F(000)**	1228	1100	1440
**index ranges**	-12<h<11	-9<h<8	-11<h<13
	-24<k<25	-15<k<15	-14<k<12
	-17<l<18	-23<l<23	-31<l<27
**data/restraints/params**	5726/0/270	6987/260/523	6325/0/313
**GOF (F2)**	1.045	1.017	1.035
**R1, wR2 [I > 2σ(I)]**	0.0290, 0.0795	0.0352, 0.0482	0.0258, 0.0613
**R1, wR2 (all data)**	0.0341, 0.0827	0.0713, 0.0542	0.0299, 0.0630

Table 2. Selected Bond Lengths (Å) for All of the Compounds

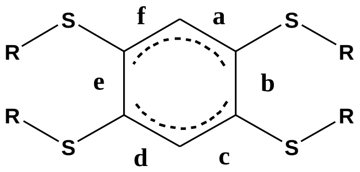

R=C_2H_5, C_3H_7

	**$TPB^{\bullet+}$** in **$TPB^{\bullet+}SbF_6^-$,**	**$TEB^{\bullet+}$** in **$TEB^{\bullet+}SbF_6^-$**	**$TEB^{\bullet+}$** in **$2TEB^{\bullet+}(TEB)2SbF_6^-$**	**TEB** in **$2TEB^{\bullet+}(TEB)2SbF_6^-$**
**a**	1.3935(3)	1.3873(1)	1.3926(3)	*1.3951(3)*
**b**	1.4467(3)	1.4400(1)	1.4454(3)	*1.4104(3)*
**c**	1.3825(3)	1.3897(1)	1.3894(3)	*1.3939(3)*
**d**	1.3885(3)	1.4078(1)	1.3952(3)	*1.3951(3)*
**e**	1.4461(3)	1.4477(1)	1.4380(3)	*1.4104(3)*
**f**	1.3920(3)	1.3874(1)	1.3917(3)	*1.3939(3)*

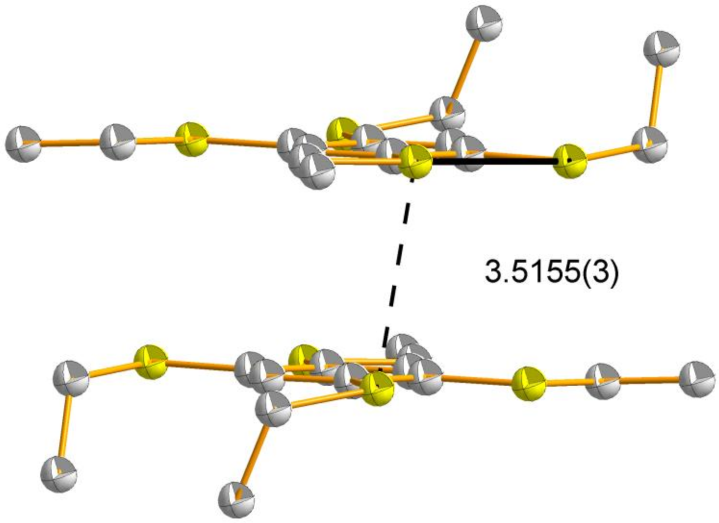

Figure 1. Structure of the stack of TEB$^{\bullet +}$ and the intermolecular interactions within the stack (distance [Å], hydrogen atoms are not shown).

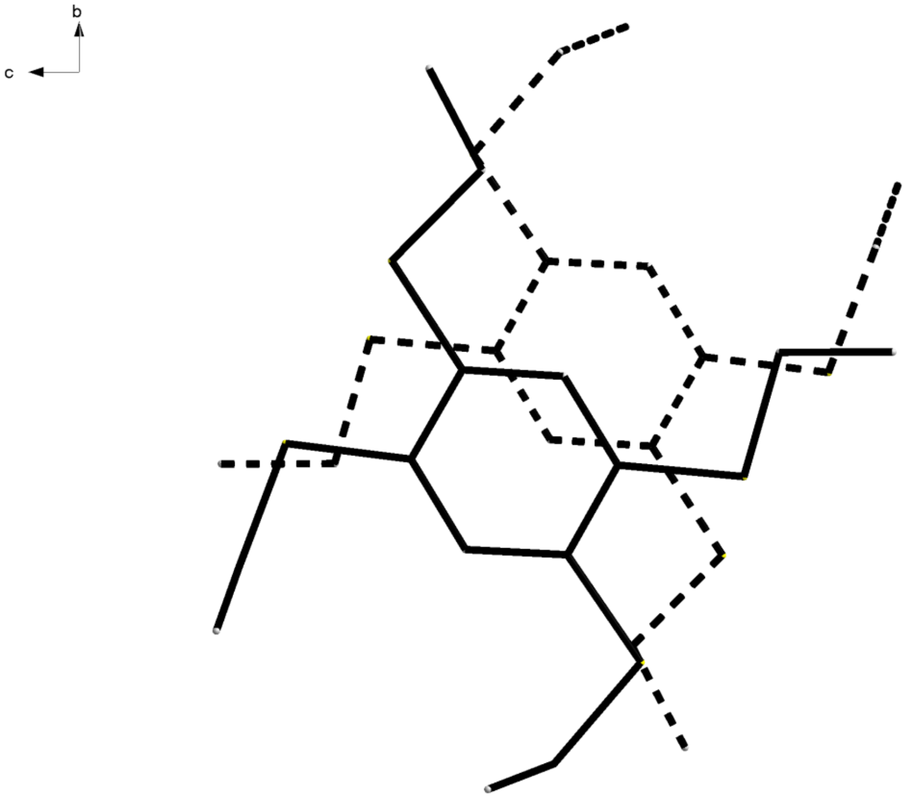

Figure 2. A stereoview of the crystal structure of TEB$^{\bullet +}$ dimmer from *a* axis.

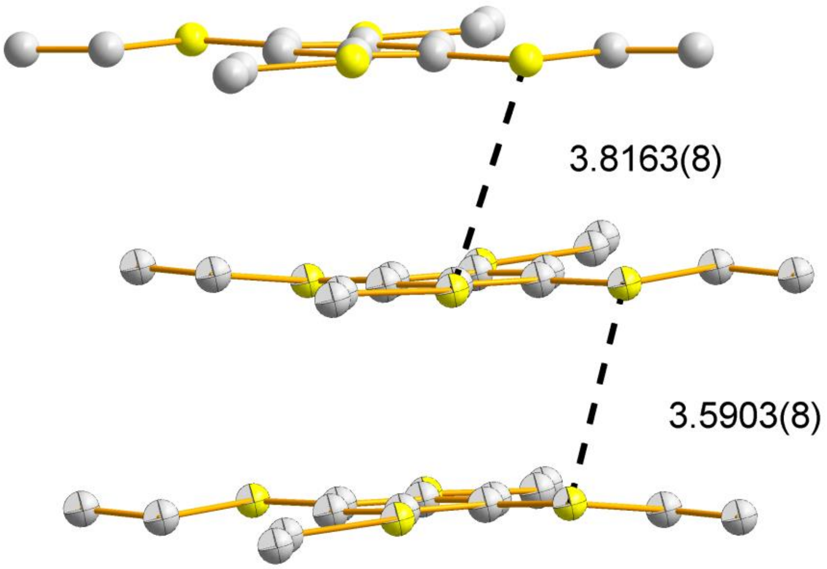

Figure 3. Structure of the stack of 2TEB$^{\bullet+}$(TEB) (distance [Å], hydrogen atoms are not shown).

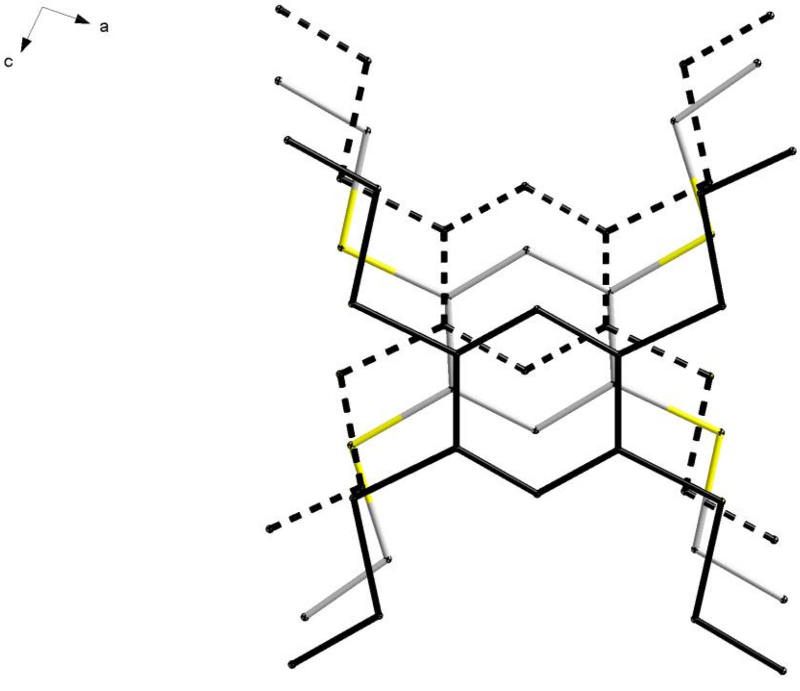

Figure 4. A stereoview of the crystal structure of 2TEB$^{\bullet+}$(TEB) from *b* axis.

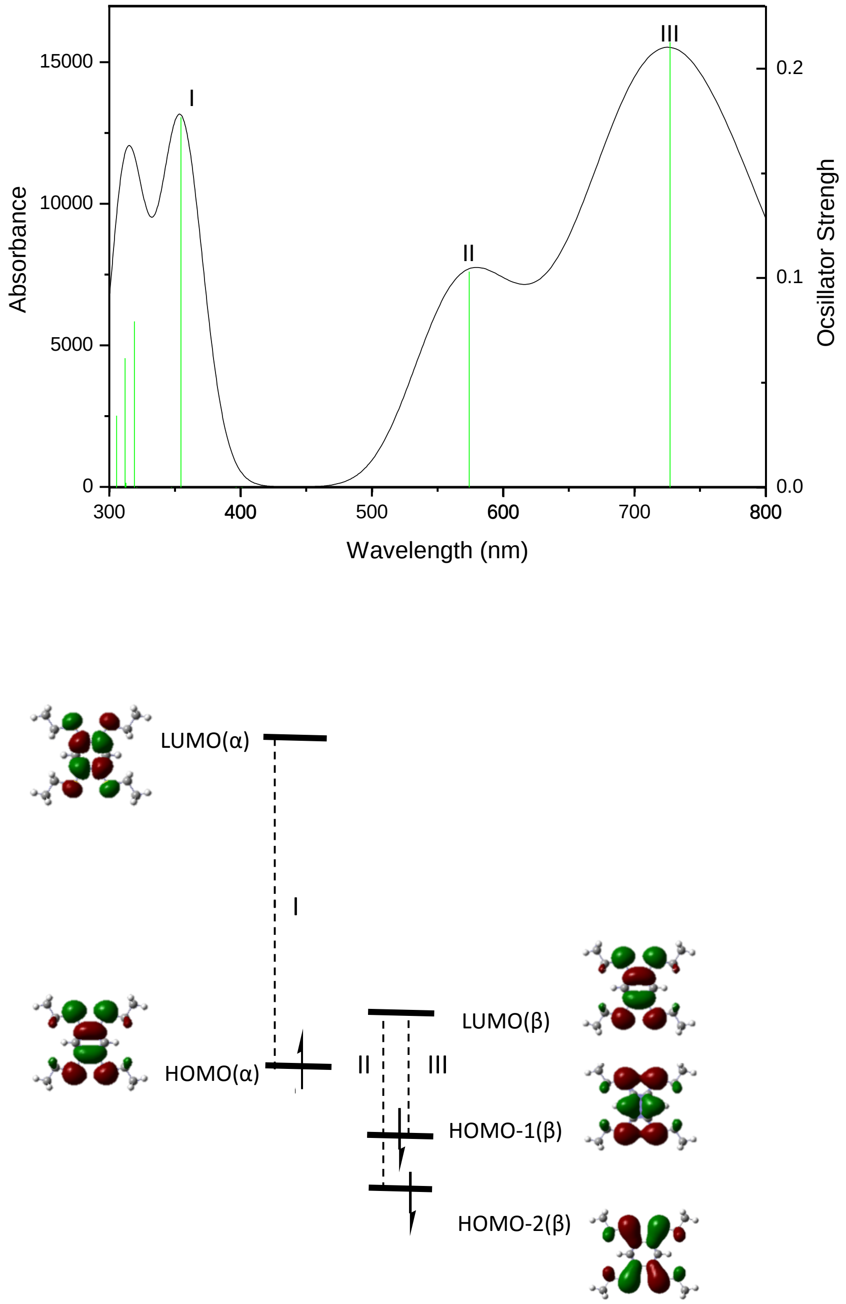

Figure 5. Top) UV absorption spectrum of $TEB^{\bullet+}$calculated at the (U)B3LYP/6-31+G(d,p) level. The spectra were simulated by using a Lorentzian convolution with 500 cm^{-1} half-widths; Bottom) Frontier molecular orbitals and electronic transitions of $TEB^{\bullet+}$.

The calculated UV absorption spectrum of $2TEB^{\bullet+}(TEB)2SbF_6^-$ is not shown here because the position of peaks of neutral TEB are all below 350 nm, which has little influence on the main absorption peaks in UV-vis spectrum.
